# Association of Primary Care Clinic Appointment Time With Clinician Ordering and Patient Completion of Breast and Colorectal Cancer Screening

**DOI:** 10.1001/jamanetworkopen.2019.3403

**Published:** 2019-05-10

**Authors:** Esther Y. Hsiang, Shivan J. Mehta, Dylan S. Small, Charles A. L. Rareshide, Christopher K. Snider, Susan C. Day, Mitesh S. Patel

**Affiliations:** 1 School of Medicine, Johns Hopkins University, Baltimore, Maryland; 2 Wharton School, University of Pennsylvania, Philadelphia; 3 Perelman School of Medicine, University of Pennsylvania, Philadelphia; 4 Penn Medicine Nudge Unit, University of Pennsylvania, Philadelphia; 5 Crescenz Veterans Affairs Medical Center, Philadelphia, Pennsylvania

## Abstract

**Importance:**

As the clinic day progresses, clinicians may fall behind schedule and experience decision fatigue. However, the association of time of day with cancer screening rates is unknown.

**Objective:**

To evaluate the association of primary care clinic appointment time with clinician ordering and patient completion of breast and colorectal cancer screening.

**Design, Setting, and Participants:**

Retrospective, quality improvement study of 33 primary care practices in Pennsylvania and New Jersey from September 1, 2014, to August 31, 2016. Participants included adults eligible for breast or colorectal cancer screening. Data analysis was conducted from April 24, 2018, to November 8, 2018.

**Exposures:**

Clinic appointment time during each patient’s first primary care physician visit in the study period.

**Main Outcomes and Measures:**

Primary outcome was clinician ordering of the screening test during the visit. Secondary outcome was patient completion of the tests within 1 year of the visit.

**Results:**

Among the 19 254 patients eligible for breast cancer screening, the mean (SD) age was 60.2 (6.9) years; 19 254 (100%) were female, 11 682 (60.7%) were white, and 5495 (28.5%) were black. Screening test order rates were highest at 8 am at 63.7%, decreased throughout the morning to 48.7% at 11 am, increased to 56.2% at noon, and then decreased to 47.8% at 5 pm (adjusted odds ratio [OR] for overall trend, 0.94; 95% CI, 0.93-0.96; *P* < .001). Trends in screening test completion rates were similar beginning at 33.2% at 8 am and decreasing to 17.8% at 5 pm (adjusted OR, 0.95; 95% CI, 0.94-0.97; *P* < .001). Among the 33 468 patients eligible for colorectal cancer screening, the mean (SD) age was 59.6 (7.4) years; 18 672 (55.8%) were female, 22 157 (66.2%) were white, and 7296 (21.8%) were black. Screening test order rates were 36.5% at 8 am, decreased to 31.3% by 11 am, increased at noon to 34.4%, and then decreased to 23.4% at 5 pm (adjusted OR, 0.94; 95% CI, 0.93-0.95; *P* < .001). Trends in screening test completion rates were similar beginning at 28.0% at 8 am and decreasing to 17.8% at 5 pm (adjusted OR, 0.97; 95% CI, 0.96-0.98; *P* < .001).

**Conclusions and Relevance:**

Clinician ordering of cancer screening tests significantly decreased as the clinic day progressed. Patient completion of cancer screening tests within 1 year of the visit was also lower as the primary care appointment time was later in the day. Future interventions targeting improvements in cancer screening should consider how time of day may influence these behaviors.

## Key Points

**Question:**

Are breast and colorectal cancer screening rates associated with the time of day a patient visits the primary care clinician?

**Findings:**

In this quality improvement study analysis of 33 primary care practices including 19 254 patients eligible for breast cancer screening and 33 468 patients eligible for colorectal cancer screening, both clinician ordering and patient completion of cancer screening tests decreased as the time of day progressed.

**Meaning:**

Patients with primary care clinic appointment times later in the day were less likely to be ordered for and receive guideline recommended cancer screening.

## Teaser

This quality improvement study evaluates the association of primary care clinic appointment time with clinician ordering and patient completion of breast and colorectal cancer screening.

## Introduction

Cancer is a leading cause of mortality in the United States.^1^ Appropriate cancer screening can be effective in decreasing both morbidity and mortality by detecting and treating cancers at an earlier stage.^2^ However, underuse of cancer screening tests is common.^2,3,4,5,6,7^ For example, the Centers for Disease Control and Prevention estimates that among patients who meet guideline recommendations, approximately 37% of adults have not been screened for colorectal cancer, and 28% of women have not been screened for breast cancer.^8^

The US Preventive Services Task Force guidelines recommend that primary care physicians (PCPs) offer breast and colorectal cancer screening to eligible patients during clinic visits.^9,10^ However, as the clinic day progresses, PCPs may fall behind schedule and this may result in shorter and more rushed interactions with patients scheduled later in the day. As the clinic day progresses, PCPs may also face decision fatigue, which is defined as the depletion of self-control and active initiative that results from the cumulative burden of decision making.^11^ These tendencies may lead to suboptimal care for patients with clinic appointment times later in the day. For example, in prior work we found that influenza vaccination rates began around 44% in the morning but then steadily decreased to 32% by the end of the day.^12^ These patterns have also been found to exist for other behaviors. Evidence indicates that later in the day, there are higher rates of inappropriate antibiotic prescriptions by PCPs,^13^ higher rates of opioid prescribing for back pain by PCPs,^14^ and lower rates of appropriate handwashing among clinicians during the end of hospital shifts.^15^

To our knowledge, variations in clinician ordering of cancer screening tests by clinic appointment time have not been well examined. Moreover, the downstream effect of whether the patient completes cancer screening based on clinic appointment time is unknown. In this study, our objective was to evaluate the association of primary care clinic appointment time with clinician ordering and patient completion of breast and colorectal cancer screening.

## Methods

This study was approved by the University of Pennsylvania institutional review board, which granted a waiver of informed consent because the study posed minimal risk and because it was infeasible given the retrospective study design. This study followed the Standards for Quality Improvement Reporting Excellence (SQUIRE) reporting guideline.

### Setting and Participants

The sample comprised patient visits from 33 primary care practice sites at the University of Pennsylvania Health System. These practice sites were located in Pennsylvania and New Jersey and included both internal medicine and family medicine clinicians (eTable 1 in the Supplement). During the study period (September 1, 2014, to August 31, 2016), we evaluated each patient’s first new or return visit with a PCP at 1 of the practice sites. Similar to prior work,^12^ other types of visits (eg, acute or sick visits) were excluded because preventive screening may be less likely to be discussed and instead deferred to the next visit, and patients were excluded if they changed PCPs during the study period (eFigure 1 and eFigure 2 in the Supplement).

Data from the electronic health record were used to include patients who were due for either breast or colorectal cancer screening based on the US Preventive Services Task Force guidelines.^9,10^ For breast cancer screening, this included women between ages 50 and 74 years. For colorectal cancer screening, this included adults between ages 50 and 75 years. Using health maintenance information and data in the electronic health record, we looked back up to 10 years to evaluate prior patient interactions and screenings to determine eligibility.

### Data

Similar to prior work,^7,12^ Clarity, an EPIC reporting database, was used to obtain data on patients, clinic visits, and cancer screening tests. Data on patients included demographic characteristics, insurance, comorbidities, PCP, and presence of cancer screening test results. Data on clinic visits included date, appointment time, practice site, visit type, and presence of an order for cancer screening tests. Breast cancer screening could be completed by mammography. Colorectal cancer screening could be completed by colonoscopy, sigmoidoscopy, fecal immunochemical test, fecal occult blood test, or multitargeted stool DNA test (Cologuard). Electronic health record codes used to classify screening tests are available in eTable 2 in the Supplement. Household income level was obtained using US Census data on median household income based on zip code. Health insurance claims data were not available for this study.

### Outcome Measures

The primary outcome was clinician ordering of the screening test during the primary care visit. The secondary outcome was patient completion of the test within 1 year of the primary care visit.

### Statistical Analysis

To evaluate the outcomes by clinic appointment time, appointment times from 8:00 am to 5:59 pm were grouped by the hour (eg, appointments for 8 am, 8:15 am, 8:30 am, and 8:45 am were grouped to 8 am). In the adjusted analysis of patient-visit level data, we used PROC LOGISTIC in SAS to fit a conditional logistic regression, where the conditioning is on PCPs.^16^ We used a conditional logistic regression model because clinicians differ in the times of day they interact with patients and this model stratifies the analysis by clinician so that the model evaluates each individual clinician. This prevents a scenario whereby clinicians only who practice in the afternoon are compared with clinicians who only practice in the morning. As a sensitivity analysis, we also fit a generalized estimating equation model with a logit link and an independence correlation structure using PCP as the clustering unit without conditioning on PCP.

The models were adjusted for patient demographics (age, sex, race and ethnicity, and household income), insurance, Charlson comorbidity index,^17^ clinic visit type (new or return), fixed effects by practice site, year and calendar month, and a covariate for each appointment hour. To obtain an adjusted overall trend of screening tests as the clinic day progressed, the same models were fit, but instead of covariates for each appointment hour, a continuous variable for appointment time was used (from 8 am to 5 pm). Two-sided hypothesis tests used a significance level of .05; all analyses were conducted in SAS, version 9.4 (SAS Institute Inc).

## Results

The sample eligible for breast cancer screening comprised 19 254 patients with a mean (SD) age of 60.2 (6.9) years; 19 254 (100.0%) were female, 11 682 (60.7%) were white, and 5495 (28.5%) were black (Table 1). The sample eligible for colorectal cancer screening comprised 33 468 patients with a mean (SD) age of 59.6 (7.4) years; 18 672 (55.8%) were female, 22 157 (66.2%) were white, and 7296 (21.8%) were black. Characteristics of patients for both samples were similar across clinic appointment times throughout the day (eTable 3 and eTable 4 in the Supplement).

**Table 1.** Sample Characteristics of Patients Visiting With Their Primary Care Physician and Eligible for Cancer Screening

Characteristic	No. (%)	
	Eligible for Breast Cancer Screening	Eligible for Colorectal Cancer Screening
Patients, No.	19 254	33 468
Age, mean (SD), y	60.2 (6.9)	59.6 (7.4)
Female	19 254 (100.0)	18 672 (55.8)
Race/ethnicity		
White non-Hispanic	11 682 (60.7)	22 157 (66.2)
Black non-Hispanic	5495 (28.5)	7296 (21.8)
Asian	563 (2.9)	967 (2.9)
Hispanic	434 (2.3)	965 (2.9)
Other or unknown	1080 (5.6)	2083 (6.2)
Insurance		
Private	12 392 (64.4)	22 288 (66.6)
Medicare	5527 (28.7)	9003 (26.9)
Medicaid	1335 (6.9)	2177 (6.5)
Annual household income, $^a^		
<50 000	6228 (32.3)	8865 (26.5)
50 000-100 000	9873 (51.3)	18 551 (55.4)
>100 000	2933 (15.2)	5622 (16.8)
Missing	220 (1.1)	430 (1.3)
Charlson comorbidity index, median (IQR)	0 (0-1)	0 (0-1)

Abbreviation: IQR, interquartile range.

^a^ Annual household income was linked to each patient using the US Census data on median household income based on zip code.

**Figure 1.**
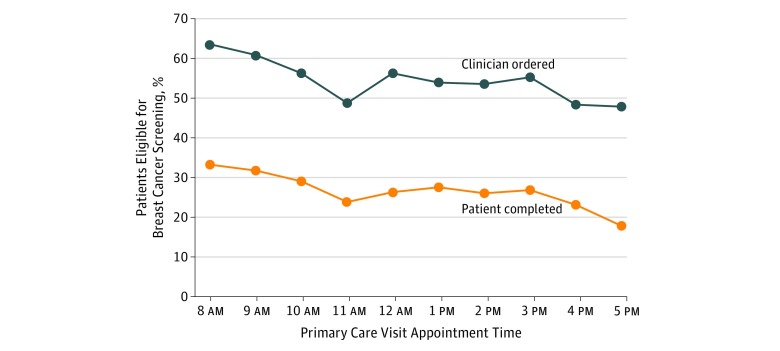
Breast Cancer Screening Order and Completion Rates by Clinic Appointment Time Unadjusted data are from September 1, 2014, to August 31, 2016, and based on each patient’s first visit with the primary care physician. Data on order rates represent the day of the primary care visit. Data on completion rates represent a 1-year follow-up from the visit. Clinic appointment times are grouped by the start of each hour (eg, 8:15 am and 8:30 am were grouped to 8 am).

Figure 1 displays the unadjusted rates of clinician ordering and patient completion of breast cancer screening from 8 am to 5 pm. Clinician order rates were 63.7% at 8 am, decreased to 48.7% by 11 am, increased at noon to 56.2%, and then remained steady until declining to approximately 47.8% at 4 pm and 5 pm. Trends in patient completion rates were similar, beginning at 33.2% at 8 am, decreasing to 23.8% at 11 am, increasing at noon to 26.3%, then remaining steady until declining at 4 pm, and decreasing to 17.8% at 5 pm.

Relative to 8 am, the adjusted odds ratios (OR) of clinician ordering and patient completion of breast cancer screening was significantly lower for each hour from 10 am to 5 pm (Table 2). The adjusted overall time trend decreased significantly for clinician ordering (adjusted OR, 0.94; 95% CI, 0.93-0.96; *P* < .001) and patient completion (adjusted OR, 0.95; 95% CI, 0.94-0.97; *P* < .001).

**Table 2.** Adjusted Odds of Breast Cancer Screening

Primary Care Appointment Time	Clinician Ordering of Screening Test Relative to 8 AM Appointment Time		Patient Completion of Screening Test Relative to 8 am Appointment Time	
	Adjusted OR (95% CI)^a^	*P* Value	Adjusted OR (95% CI)^a^	*P* Value
8 am	1 [Reference]	NA	1 [Reference]	NA
9 am	0.89 (0.78-1.01)	.06	0.91 (0.81-1.03)	.14
10 am	0.73 (0.64-0.83)	<.001	0.81 (0.71-0.91)	<.001
11 am	0.55 (0.48-0.63)	<.001	0.64 (0.55-0.73)	<.001
12 pm	0.60 (0.49-0.75)	<.001	0.63 (0.51-0.78)	<.001
1 pm	0.64 (0.56-0.73)	<.001	0.73 (0.62-0.83)	<.001
2 pm	0.65 (0.57-0.75)	<.001	0.71 (0.62-0.81)	<.001
3 pm	0.68 (0.59-0.78)	<.001	0.78 (0.68-0.89)	<.001
4 pm	0.49 (0.41-0.58)	<.001	0.60 (0.50-0.71)	<.001
5 pm	0.53 (0.42-0.69)	<.001	0.49 (0.37-0.65)	<.001
Overall time trend^b^	0.94 (0.93-0.96)	<.001	0.95 (0.94-0.97)	<.001

Abbreviations: NA, not applicable; OR, odds ratio.

^a^ Adjusted ORs represent the relative odds of screening for each hour after 8 am (reference group). Appointment times are grouped by the start of the hour (eg, 8:15 am and 8:30 am were grouped into 8 am).

^b^ Overall time trend uses an adjusted model with a continuous variable for appointment time with 1 equal to 8 am and 9 equal to 4 pm. The ORs represents the relative odds of screening for each incremental 1-hour period. For example, an OR of 0.95 can be interpreted at 5% lower odds per hour for each hour after 8 am.

**Figure 2.**
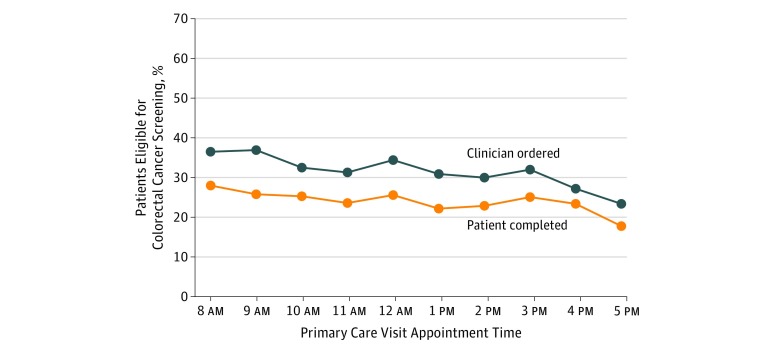
Colorectal Cancer Screening Order and Completion Rates by Clinic Appointment Time Unadjusted data are from September 1, 2014, to August 31, 2016, and based on each patient’s first visit with their primary care physician. Data on order rates represent the day of the primary care visit. Data on completion rates represent a 1-year follow-up from the visit. Clinic appointment times are grouped by the start of each hour (eg, 8:15 am and 8:30 am were grouped to 8 am).

Figure 2 displays the unadjusted rates of clinician ordering and patient completion of colorectal cancer screening from 8 am to 5 pm. Clinician order rates were 36.5% at 8 am and 36.9% at 9 am, decreased to 31.3% at 11 am, and then increased to 34.4% at noon. In the afternoon, clinician order rates were between 30% and 32% until decreasing to 27.2% at 4 pm and 23.4% at 5 pm. Trends in patient completion rates were similar, beginning at 28.0%, decreasing to 23.6% at 11 am, increasing to 25.6% at noon, and decreasing to 17.8% at 5 pm. The distribution of tests used to meet colorectal cancer screening were similar across different times of the day (eTable 5 in the Supplement).

Relative to 8 am, the adjusted OR of clinician ordering of colorectal cancer screening was significantly lower for each hour from 10 am to 5 pm and the adjusted OR of patient completion was lower for each hour from 9 am to 5 pm (Table 3). The adjusted overall time trend decreased significantly for clinician ordering (adjusted OR, 0.94; 95% CI, 0.93-0.95; *P* < .001) and patient completion (adjusted OR, 0.97; 95% CI, 0.96-0.98; *P* < .001).

**Table 3.** Adjusted Odds of Colorectal Cancer Screening

Primary Care Appointment Time	Clinician Ordering of Screening Test Relative to 8 am Appointment Time		Patient Completion of Screening Test Relative to 8 am Appointment Time	
	Adjusted OR (95% CI)^a^	*P* Value	Adjusted OR (95% CI)^a^	*P* Value
8 am	1 [Reference]	NA	1 [Reference]	NA
9 am	0.91 (0.83-1.01)	.08	0.86 (0.78-0.95)	.002
10 am	0.78 (0.71-0.87)	<.001	0.88 (0.79-0.96)	.007
11 am	0.64 (0.57-0.72)	<.001	0.77 (0.69-0.86)	<.001
12 pm	0.56 (0.47-0.67)	<.001	0.76 (0.64-0.90)	.002
1 pm	0.68 (0.60-0.76)	<.001	0.72 (0.65-0.81)	<.001
2 pm	0.64 (0.58-0.72)	<.001	0.73 (0.66-0.81)	<.001
3 pm	0.66 (0.59-0.74)	<.001	0.80 (0.72-0.89)	<.001
4 pm	0.50 (0.43-0.57)	<.001	0.74 (0.65-0.85)	<.001
5 pm	0.54 (0.44-0.67)	<.001	0.60 (0.48-0.74)	<.001
Overall time trend^b^	0.94 (0.93-0.95)	<.001	0.97 (0.96-0.98)	<.001

Abbreviations: NA, not applicable; OR, odds ratio.

^a^ Adjusted ORs represent the relative odds of screening for each hour after 8 am (reference group). Appointment times are grouped by the start of the hour (eg, 8:15 am and 8:30 am were grouped into 8 am).

^b^ Overall time trend uses an adjusted model with a continuous variable for appointment time with 1 equal to 8 am and 9 equal to 4 pm. The ORs represents the relative odds of screening for each incremental 1-hour period. For example, an OR of 0.95 can be interpreted at 5% lower odds per hour for each hour after 8 am.

Findings were similar in the adjusted sensitivity models for both patient samples. Regression tables for both the conditional logistic models and the adjusted sensitivity models are available in eTables 6-13 in the Supplement.

## Discussion

Among a network of 33 primary care practices, ordering of breast and colorectal cancer screening rates decreased as the clinic day progressed, most notably toward the end of the morning and afternoon shifts. A 1-year follow-up found that completion of these cancer screening tests had similar patterns. To our knowledge, this is the first study of its kind to demonstrate that primary care clinic appointment time is associated with both ordering and completion of screening for breast and colorectal cancer.

These findings expand our understanding of how time of day may influence medical decision making for cancer screening in several ways. First, there may be clinician and patient factors affecting the ordering of cancer screening tests. As each shift progresses, clinicians may fall behind schedule. The tendency may lead to shorter interactions with the patient at the end of the morning and afternoon shifts. In these situations, cancer screening may not be discussed or may be deferred to the future. As the overall clinic day progresses, clinicians may face decision fatigue, defined as the depletion of self-control and active initiative that results from the cumulative burden of decision making.^11^ In other words, as the day goes on, clinicians may be less likely to discuss cancer screening with patients simply because they have already done this (and made other decisions) a number of times. As patients earlier in the day decline screening despite the clinician’s recommendation, it could influence how likely the clinician is to bring up the topic later in the day with a different patient. In our analysis, no observable differences were found between characteristics of patients who had visits at different times of day. However, it could be the case that patients who see their clinician later in the day want to leave sooner and decline a discussion about cancer screening. Variations in care have been demonstrated for other aspects of medical decision making in primary care, including our prior evaluation that found lower influenza vaccination rates at the end of the day.^12^ Other studies have found higher rates of inappropriate antibiotic prescribing and opioid prescribing later in the day.^13,14^ In each of these studies, behaviors improved slightly after lunch (a short break for most clinicians), as was the case in the current study.

Second, we found that the pattern of cancer screening test completion during a 1-year follow-up was similar to the pattern of order rates. This indicates that decisions made during a single PCP visit may have a lasting effect on patient behavior. Clinicians may decide to defer discussion of cancer screening or other guideline-recommended care to future visits, and these findings indicate that this could potentially result in suboptimal care. One important difference between cancer screening and our prior study on influenza vaccination is that completing screening requires steps outside of the primary care visit. The screening tests must be scheduled with another department (eg, radiology or gastroenterology) and then the patient must show up to complete the test. To improve completion rates, this may need to involve interventions outside of the primary care visit.

Future work could be conducted to further understand the existing behaviors identified in this study such as evaluating the relative contributions of clinician vs patient factors on variations in ordering of cancer screening tests, as well as other factors associated with patient completion of screening tests. Future work could also be focused on ways to improve these behaviors patterns. For example, our prior work has shown how a nudge in the electronic health record can improve influenza vaccination rates across all times of day,^11^ but further work is still needed to understand how to lower the decline in care over the course of the day.

### Limitations

This study had limitations. First, this is an observational study and is susceptible to unmeasured confounders. However, we have adjusted for patient and practice-based characteristics as available and the findings in this cancer screening study have been found for other health behaviors in primary care settings. Second, this study was conducted at a single health system, which limits generalizability. However, we included 33 practices from 2 states that were in both urban and more rural settings. Third, variations in test ordering and completion may be due to clinician and patient factors, which, while we were unable to disentangle in this study, were adjusted for in the analysis. Fourth, we did not have access to insurance claims data and therefore were not able to capture test ordering or completion that occurred outside of this health system.

## Conclusions

Clinician ordering of breast and colorectal screening tests decreased significantly as the clinic day progressed. Patient completion of these cancer screening tests within 1 year of the visit was also significantly lower as the primary care appointment time was later in the day. Future interventions targeting improvements in cancer screening should consider how time of day influences these behaviors.

## Notes

Supplement. **eTable 1.** Practice Site Location and Specialty **eTable 2.** Electronic Health Record Codes Used to Classify Screening Tests **eTable 3.** Sample Characteristics of Patients Visiting With Their Primary Care Physician and Eligible for Breast Cancer Screening by Hour **eTable 4.** Sample Characteristics of Patients Visiting With Their Primary Care Physician and Eligible for Colorectal Cancer Screening by Hour **eTable 5.** Distribution of Colorectal Cancer Screening Tests Across Different Times of Day **eTable 6.** Regression Table for Conditional Logistic Clinic Appointment Time Model With Hourly Appointment Time Variables for Breast Cancer Screening **eTable 7.** Regression Table for Conditional Logistic Clinic Appointment Time Model With Continuous Appointment Time Variables for Breast Cancer Screening **eTable 8. **Regression Table for Conditional Logistic Clinic Appointment Time Model With Hourly Appointment Time Variables for Colorectal Cancer Screening **eTable 9.** Regression Table for Conditional Logistic Clinic Appointment Time Model With Continuous Appointment Time Variables for Colorectal Cancer Screening **eTable 10**. Regression Table for Generalized Estimated Equations Model Clustered on Primary Care Physician With Hourly Appointment Time Variables for Breast Cancer Screening **eTable 11.** Regression Table for Generalized Estimated Equations Model Clustered on Primary Care Physician With Continuous Appointment Time Variables for Breast Cancer Screening **eTable 12.** Regression Table for Generalized Estimated Equations Model Clustered Around Primary Care Physician With Hourly Appointment Time Variables for Colorectal Cancer Screening **eTable 13.** Regression Table for Generalized Estimated Equations Model Clustered Around Primary Care Physician With Continuous Appointment Time Variables for Colorectal Cancer Screening **eFigure 1.** Exclusions for the Patient Sample in Breast Cancer Screening **eFigure 2.** Exclusions for the Patient Sample in Colorectal Cancer Screening
