# Supplementary material for: Association of Primary Care Clinic Appointment Time With Clinician Ordering and Patient Completion of Breast and Colorectal Cancer Screening
Source: JAMA Netw Open. 2019 May 10;2(5):e193403. doi: 10.1001/jamanetworkopen.2019.3403 (PMC6512279; doi:10.1001/jamanetworkopen.2019.3403)
Supplement: Supplement. — eTable 1. Practice Site Location and Specialty eTable 2. Electronic Health Record Codes Used to Classify Screening Tests eTable 3. Sample Characteristics of Patients Visiting With Their Primary Care Physician and Eligible for Breast Cancer Screening by Hour eTable 4. Sample Characteristics of Patients Visiting With Their Primary Care Physician and Eligible for Colorectal Cancer Screening by Hour eTable 5. Distribution of Colorectal Cancer Screening Tests Across Different Times of Day eTable 6. Regression Table for Conditional Logistic Clinic Appointment Time Model With Hourly Appointment Time Variables for Breast Cancer Screening eTable 7. Regression Table for Conditional Logistic Clinic Appointment Time Model With Continuous Appointment Time Variables for Breast Cancer Screening eTable 8. Regression Table for Conditional Logistic Clinic Appointment Time Model With Hourly Appointment Time Variables for Colorectal Cancer Screening eTable 9. Regression Table for Conditional Logistic Clinic Appointment Time Model With Continuous Appointment Time Variables for Colorectal Cancer Screening eTable 10. Regression Table for Generalized Estimated Equations Model Clustered on Primary Care Physician With Hourly Appointment Time Variables for Breast Cancer Screening eTable 11. Regression Table for Generalized Estimated Equations Model Clustered on Primary Care Physician With Continuous Appointment Time Variables for Breast Cancer Screening eTable 12. Regression Table for Generalized Estimated Equations Model Clustered Around Primary Care Physician With Hourly Appointment Time Variables for Colorectal Cancer Screening eTable 13. Regression Table for Generalized Estimated Equations Model Clustered Around Primary Care Physician With Continuous Appointment Time Variables for Colorectal Cancer Screening eFigure 1. Exclusions for the Patient Sample in Breast Cancer Screening eFigure 2. Exclusions for the Patient Sample in Colorectal Cancer Screening [file jamanetwopen-2-e193403-s001.pdf]

Supplementary Online Content

Hsiang EY, Mehta SJ, Small DS, et al. Association of primary care clinic appointment time with clinician ordering and patient completion of breast and colorectal cancer screening. *JAMA Netw Open*. 2019;2(5):e193403. doi:10.1001/jamanetworkopen.2019.3403

eTable 1. Practice Site Location and Specialty

eTable 2. Electronic Health Record Codes Used to Classify Screening Tests

eTable 3. Sample Characteristics of Patients Visiting With Their Primary Care Physician and Eligible for Breast Cancer Screening by Hour

eTable 4. Sample Characteristics of Patients Visiting With Their Primary Care Physician and Eligible for Colorectal Cancer Screening by Hour

eTable 5. Distribution of Colorectal Cancer Screening Tests Across Different Times of Day

eTable 6. Regression Table for Conditional Logistic Clinic Appointment Time Model With Hourly Appointment Time Variables for Breast Cancer Screening

eTable 7. Regression Table for Conditional Logistic Clinic Appointment Time Model With Continuous Appointment Time Variables for Breast Cancer Screening

eTable 8. Regression Table for Conditional Logistic Clinic Appointment Time Model With Hourly Appointment Time Variables for Colorectal Cancer Screening

eTable 9. Regression Table for Conditional Logistic Clinic Appointment Time Model With Continuous Appointment Time Variables for Colorectal Cancer Screening

eTable 10. Regression Table for Generalized Estimated Equations Model Clustered on Primary Care Physician With Hourly Appointment Time Variables for Breast Cancer Screening

eTable 11. Regression Table for Generalized Estimated Equations Model Clustered on Primary Care Physician With Continuous Appointment Time Variables for Breast Cancer Screening

eTable 12. Regression Table for Generalized Estimated Equations Model Clustered Around Primary Care Physician With Hourly Appointment Time Variables for Colorectal Cancer Screening

eTable 13. Regression Table for Generalized Estimated Equations Model Clustered Around Primary Care Physician With Continuous Appointment Time Variables for Colorectal Cancer Screening

© 2019 Hsiang EY et al. *JAMA Network Open*.

eFigure 1. Exclusions for the Patient Sample in Breast Cancer Screening

eFigure 2. Exclusions for the Patient Sample in Colorectal Cancer Screening

This supplementary material has been provided by the authors to give readers additional information about their work.

© 2019 Hsiang EY et al. *JAMA Network Open*.

eTable 1. Practice Site Location and Specialty

Practice Name	Location	Specialty
Penn Family Medicine Southern Chester County	West Grove, PA	Family Medicine
Penn Family Medicine West Chester	West Chester, PA	Family Medicine
Penn Family Medicine Phoenixville	Phoenixville, PA	Family Medicine
Penn Family Care	Philadelphia, PA	Family Medicine
Penn Family Medicine Chestnut Hill	Philadelphia, PA	Family Medicine
Penn Family Medicine University City	Philadelphia, PA	Family Medicine
Penn Family Medicine Limerick	Limerick, PA	Family Medicine
Penn Family Medicine Kennett	Kennett Square, PA	Family Medicine
Penn Family Medicine New Garden	Kennett Square, PA	Family Medicine
Penn Family Medicine Unionville	Kennett Square, PA	Family Medicine
Penn Family Medicine Gibbsboro	Gibbsboro, NJ	Family Medicine
Penn Primary Care and Integrative Medicine Whiteland	Exton, PA	Family Medicine
Penn Family Medicine Valley Forge	Berwyn, PA	Family Medicine
Penn Medicine Woodbury Heights	Woodbury Heights, NJ	Internal Medicine
Penn Internal Medicine East Marshall Street	West Chester, PA	Internal Medicine
Penn Radnor Internal Medicine	Radnor, PA	Internal Medicine
Penn Radnor Internal Medicine Geriatrics	Radnor, PA	Internal Medicine
Penn General Internal Medicine Perelman	Philadelphia, PA	Internal Medicine
Delancey Medicine Associates	Philadelphia, PA	Internal Medicine
Penn Center for Primary Care, Penn Presbyterian Medical Center	Philadelphia, PA	Internal Medicine
Penn Internal Medicine - J. Edwin Wood Clinic	Philadelphia, PA	Internal Medicine
Penn Internal Medicine Mayfair	Philadelphia, PA	Internal Medicine
Penn Internal Medicine University City	Philadelphia, PA	Internal Medicine

© 2019 Hsiang EY et al. *JAMA Network Open*.

Penn Internal Medicine – Michael Cirigliano Internal Medicine	Philadelphia, PA	Internal Medicine
Penn Presbyterian Internal Medicine	Philadelphia, PA	Internal Medicine
Penn Spruce Internal Medicine	Philadelphia, PA	Internal Medicine
Penn Internal Medicine Media	Media, PA	Internal Medicine
Penn Family and Internal Medicine Lincoln	West Chester, PA	Internal Medicine, Family Medicine
Penn Family and Internal Medicine Longwood	Kennett Square, PA	Internal Medicine, Family Medicine
Penn Family and Internal Medicine Cherry Hill	Cherry Hill, NJ	Internal Medicine, Family Medicine
Penn Medicine Bucks County	Yardley, PA	Internal Medicine, Family Medicine
Penn Medicine Rittenhouse	Philadelphia, PA	Internal Medicine, Family Medicine
Penn Medicine Bala Cynwyd	Bala Cynwyd, PA	Internal Medicine, Family Medicine

© 2019 Hsiang EY et al. *JAMA Network Open*.

eTable 2. Electronic Health Record Codes Used to Classify Screening Tests

Cancer Screening	Test	Codes
Breast Cancer Screening	Mammogram	87.36, 87.37, 77055, 77056, 77057, 77065, 77066, 77067, 111224, 111229, CADS, G0202, G0204, G0206, IMGMAM0001, IMGMAM0010, IMGMAM0012, IMGMAM0016, IMGMAM0015, IMGMAM0018, IMGMAM0021, IMGMAM0023, IMGMAM0035, IMGMAM0027, IMGMAM0037, IMGMAM0041, IMGMAM0082, MABD, MABDBU, MABDT, MALD, MALDT, MAMB, MAMS, MARD, MARDBU, MARDT, MASD, MASDBU, MASDT, MAUD, RADHX0328, RADHX1242, RADHX1266, IMGMAM0087, IMGMAM0089
Colorectal Cancer Screening	Colonoscopy	45.22, 45.23, 45.25, 45.42, 45.43, 44388, 9053, 44389, 44390, 44391, 44392, 44394, 44393, 44397, 45355, 45378, 45379, 45380, 45381, 45383, 45382, 45385, 45384, 45386, 45387, 45391, 45392, 111227, G0105, G0121, GI10, 0528F, 3000004, 44403, 44404, 45390
	FIT / FOBT	35301, 82270, 82274, 83520, 111239, C6000980, FIT, G0328, OCCBI
	Multitargeted Stool DNA Test	111239, PROC7007
	Sigmoidoscopy	45.24, 45330, 45331, 45333, 45334, 45335, 45332, 45337, 45338, 45339, 45340, 45341, 45342, 45345, G0104, 3000006, 45300, 45305, 45315, 45317, 45346, GI12

© 2019 Hsiang EY et al. *JAMA Network Open*.

eTable 3. Sample Characteristics of Patients Visiting With Their Primary Care Physician and Eligible for Breast Cancer Screening by Hour

**Characteristic**	**Overall**	**8AM**	**9AM**	**10AM**	**11AM**	**12PM**	**1PM**	**2PM**	**3PM**	**4PM**	**5PM**
Patients, No.	19254	2267	3081	2938	2062	585	2200	2646	2084	1025	366
Age, Mean (SD), y	60.2 (6.9)	59.6 (6.7)	60.1 (6.8)	60.9 (7.1)	60.7 (7.1)	61 (7)	60.6 (7)	60.4 (6.9)	59.4 (6.7)	58.8 (6.3)	58.3 (6.2)
Female, No. (%)		-	-	-	-	-	-	-	-	-	-
Race/Ethnicity, No. (%)											
White Non-hispanic	11682 (60.7)	1475 (65.1)	1809 (58.7)	1758 (59.8)	1278 (62)	377 (64.4)	1304 (59.3)	1577 (59.6)	1198 (57.5)	663 (64.7)	243 (66.4)
Black Non-hispanic	5495 (28.5)	62 (2.7)	137 (4.4)	79 (2.7)	49 (2.4)	18 (3.1)	62 (2.8)	60 (2.3)	59 (2.8)	26 (2.5)	11 (3.0)
Asian	563 (2.9)	544 (24)	862 (28.0)	893 (30.4)	579 (28.1)	140 (23.9)	640 (29.1)	814 (30.8)	668 (32.1)	267 (26.0)	88 (24.0)
Hispanic	434 (2.3)	64 (2.8)	71 (2.3)	64 (2.2)	32 (1.6)	4 (0.7)	66 (3.0)	66 (2.5)	41 (2.0)	17 (1.7)	9 (2.5)
Other/Unknown	1080 (5.6)	122 (5.4)	202 (6.6)	144 (4.9)	124 (6.0)	46 (7.9)	128 (5.8)	129 (4.9)	118 (5.7)	52 (5.1)	15 (4.1)
Insurance, No. (%)											
Private	12392 (64.4)	1696 (74.8)	2009 (65.2)	1709 (58.2)	1217 (59.0)	374 (63.9)	1302 (59.2)	1592 (60.2)	1403 (67.3)	781 (76.2)	309 (84.4)
Medicare	5527 (28.7)	483 (21.3)	855 (27.8)	1001 (34.1)	690 (33.5)	194 (33.2)	733 (33.3)	831 (31.4)	515 (24.7)	181 (17.7)	44 (12.0)
Medicaid	1335 (6.9)	88 (3.9)	217 (7.0)	228 (7.8)	155 (7.5)	17 (2.9)	165 (7.5)	223 (8.4)	166 (8.0)	63 (6.1)	13 (3.6)

© 2019 Hsiang EY et al. *JAMA Network Open*.

Annual Household Income, No. (%)*											
<$50,000	6228 (32.3)	588 (25.9)	1017 (33)	972 (33.1)	638 (30.9)	169 (28.9)	709 (32.2)	959 (36.2)	771 (37.0)	305 (29.8)	100 (27.3)
$50,000 to $100,000	9873 (51.3)	1278 (56.4)	1557 (50.5)	1498 (51.0)	1043 (50.6)	294 (50.3)	1118 (50.8)	1300 (49.1)	1004 (48.2)	554 (54.0)	227 (62.0)
>$100,000	2933 (15.2)	367 (16.2)	473 (15.4)	431 (14.7)	366 (17.7)	115 (19.7)	343 (15.6)	354 (13.4)	285 (13.7)	162 (15.8)	37 (10.1)
Missing	220 (1.1)	34 (1.5)	34 (1.1)	37 (1.3)	15 (0.7)	7 (1.2)	30 (1.4)	33 (1.2)	24 (1.2)	4 (0.4)	2 (0.5)
Charlson Comorbidity Index, Median (IQR)	0 (0-1)	0 (0-1)	0 (0-1)	0 (0-2)	0 (0-2)	0 (0-1)	0 (0-1)	0 (0-1)	0 (0-1)	0 (0-1)	0 (0-1)

Abbreviations: SD, standard deviation. IQR, interquartile range.

*Annual household income was linked to each patient using the US Census data on median household income based on zip code.

© 2019 Hsiang EY et al. *JAMA Network Open*.

eTable 4. Sample Characteristics of Patients Visiting With Their Primary Care Physician and Eligible for Colorectal Cancer Screening by Hour

**Characteristic**	**Overall**	**8AM**	**9AM**	**10AM**	**11AM**	**12PM**	**1PM**	**2PM**	**3PM**	**4PM**	**5PM**
Patients, No.	33468	4293	5183	5125	3427	951	3736	4429	3732	1900	692
Age, Mean (SD), y	59.6 (7.4)	58.8 (7.2)	59.6 (7.4)	60.4 (7.5)	60.2 (7.6)	60.7 (7.6)	60 (7.5)	59.7 (7.3)	59 (7.1)	58.1 (6.8)	57.3 (6.2)
Female, No. (%)	14796 (44.2)	2178 (50.7)	2315 (44.7)	2185 (42.6)	1393 (40.6)	347 (36.5)	1618 (43.3)	1843 (41.6)	1697 (45.5)	905 (47.6)	315 (45.5)
Race/Ethnicity, No. (%)											
White Non-hispanic	22157 (66.2)	3133 (73.0)	3439 (66.4)	3307 (64.5)	2295 (67.0)	677 (71.2)	2363 (63.2)	2748 (62.0)	2377 (63.7)	1324 (69.7)	494 (71.4)
Black Non-hispanic	7296 (21.8)	104 (2.4)	196 (3.8)	145 (2.8)	75 (2.2)	23 (2.4)	103 (2.8)	125 (2.8)	126 (3.4)	53 (2.8)	17 (2.5)
Asian	967 (2.9)	686 (16.0)	1077 (20.8)	1216 (23.7)	755 (22.0)	184 (19.3)	906 (24.3)	1121 (25.3)	875 (23.4)	358 (18.8)	118 (17.1)
Hispanic	965 (2.9)	134 (3.1)	158 (3.0)	158 (3.1)	79 (2.3)	6 (0.6)	128 (3.4)	143 (3.2)	96 (2.6)	50 (2.6)	13 (1.9)
Other/Unknown	2083 (6.2)	236 (5.5)	313 (6.0)	299 (5.8)	223 (6.5)	61 (6.4)	236 (6.3)	292 (6.6)	258 (6.9)	115 (6.1)	50 (7.2)
Insurance, No. (%)											
Private	22288 (66.6)	3277 (76.3)	3445 (66.5)	3089 (60.3)	2117 (61.8)	629 (66.1)	2280 (61.0)	2768 (62.5)	2587 (69.3)	1508 (79.4)	588 (85.0)
Medicare	9003 (26.9)	865 (20.1)	1415 (27.3)	1671 (32.6)	1084 (31.6)	287 (30.2)	1167 (31.2)	1280 (28.9)	859 (23.0)	300 (15.8)	75 (10.8)
Medicaid	2177 (6.5)	151 (3.5)	323 (6.2)	365 (7.1)	226 (6.6)	35 (3.7)	289 (7.7)	381 (8.6)	286 (7.7)	92 (4.8)	29 (4.2)

© 2019 Hsiang EY et al. *JAMA Network Open*.

Annual Household Income, No. (%)*											
<$50,000	8865 (26.5)	801 (18.7)	1395 (26.9)	1382 (27)	933 (27.2)	254 (26.7)	1108 (29.7)	1361 (30.7)	1065 (28.5)	423 (22.3)	143 (20.7)
$50,000 to $100,000	18551 (55.4)	2639 (61.5)	2781 (53.7)	2891 (56.4)	1801 (52.6)	507 (53.3)	1994 (53.4)	2337 (52.8)	2039 (54.6)	1121 (59)	441 (63.7)
>$100,000	5622 (16.8)	807 (18.8)	944 (18.2)	772 (15.1)	663 (19.3)	176 (18.5)	576 (15.4)	664 (15.0)	578 (15.5)	344 (18.1)	98 (14.2)
Missing	430 (1.3)	46 (1.1)	63 (1.2)	80 (1.6)	30 (0.9)	14 (1.5)	58 (1.6)	67 (1.5)	50 (1.3)	12 (0.6)	10 (1.4)
Charlson Comorbidity Index, Median (IQR)	0 (0-1)	0 (0-1)	0 (0-1)	0 (0-2)	0 (0-2)	0 (0-2)	0 (0-1)	0 (0-1)	0 (0-1)	0 (0-1)	0 (0-1)

Abbreviations: SD, standard deviation. IQR, interquartile range.

*Annual household income was linked to each patient using the US Census data on median household income based on zip code.

© 2019 Hsiang EY et al. *JAMA Network Open*.

eTable 5. Distribution of Colorectal Cancer Screening Tests Across Different Times of Day

**All Visits**					
	**Colonoscopy**	**Sigmoidoscopy**	**FIT**	**FOBT**	**Multitargeted Stool DNA Test**
Test Type, No. (%)	7760 (90.9)	23 (0.3)	248 (2.9)	500 (5.9)	10 (0.1)
**All Visits from 8 AM to 5:59 PM**					
	**Colonoscopy**	**Sigmoidoscopy**	**FIT**	**FOBT**	**Multitargeted Stool DNA Test**
Test Type, No. (%)	7468 (90.7)	23 (0.3)	241 (2.9)	493 (6)	10 (0.1)
8AM	1096 (91)	1 (0.1)	38 (3.2)	67 (5.6)	2 (0.2)
9AM	1206 (90.2)	5 (0.4)	47 (3.5)	78 (5.8)	1 (0.1)
10AM	1175 (90.6)	3 (0.2)	40 (3.1)	78 (6)	1 (0.1)
11AM	722 (89.4)	3 (0.4)	17 (2.1)	66 (8.2)	0 (0)
12PM	225 (92.6)	0 (0)	5 (2.1)	13 (5.3)	0 (0)
1PM	740 (89.2)	4 (0.5)	23 (2.8)	61 (7.3)	2 (0.2)
2PM	910 (89.7)	6 (0.6)	32 (3.2)	65 (6.4)	1 (0.1)
3PM	865 (92.5)	0 (0)	29 (3.1)	39 (4.2)	2 (0.2)
4PM	416 (93.7)	1 (0.2)	7 (1.6)	19 (4.3)	1 (0.2)
5PM	113 (91.9)	0 (0)	3 (2.4)	7 (5.7)	0 (0)

© 2019 Hsiang EY et al. *JAMA Network Open*.

eTable 6. Regression Table for Conditional Logistic Clinic Appointment Time Model With Hourly Appointment Time Variables for Breast Cancer Screening

Variable	Clinician Ordered Test		Patient Completed Test	
	Odds Ratio	*P* value	Odds Ratio	*P* value
Appointment year				
9/1/2014-8/31/2015	Ref		Ref	
9/1/2015-8/31/2016	1.12	0.0039	1.05	0.2130
Age at visit	1.01	0.0974	1.01	0.0003
Race				
White	Ref		Ref	
Asian/East Indian	1.15	0.1545	1.27	0.0109
Black	1.29	<.0001	1.20	0.0003
Hispanic	1.79	<.0001	1.68	<.0001
Other	0.95	0.4405	1.01	0.9191
Insurance				
Private	Ref		Ref	
Medicaid	0.65	<.0001	0.84	0.0160
Medicare	0.77	<.0001	0.89	0.0214
Charlson comorbidity index	0.89	<.0001	0.93	<.0001
Income				
Less than $50,000	Ref		Ref	
$50,000 to $100,000	0.97	0.6185	1.00	0.9781
Greater than $100,000	0.92	0.1982	1.04	0.5501
Missing	0.65	0.0059	0.79	0.1683
Practice site				
Penn Internal Medicine - Michael Cirigliano Internal Medicine	Ref		Ref	
Penn Family Medicine Chestnut Hill	1.00	.	1.00	.
Delancey Medicine Associates	1.00	.	1.00	.
Penn Family and Internal Medicine Lincoln	1.00	.	1.00	.
Penn Family Medicine Southern Chester County	1.00	.	1.00	.
Penn Internal Medicine University City	1.60	0.4582	3.84	0.1000
Penn General Internal Medicine Perelman	1.00	.	1.00	.
Penn Center for Primary Care, Penn Presbyterian Medical Center	1.00	.	1.00	.
Penn Radnor Internal Medicine	0.89	0.5732	0.91	0.6355

© 2019 Hsiang EY et al. *JAMA Network Open*.

Penn Radnor Internal Medicine Geriatrics	1.00	.	1.00	.
Penn Internal Medicine Media	1.00	.	1.00	.
Penn Internal Medicine Mayfair	1.44	0.389	2.00	0.2204
Penn Internal Medicine - J. Edwin Wood Clinic	1.00	.	1.00	.
Penn Family Medicine Kennett	1.00	.	1.00	.
Penn Family Care	1.00	.	1.00	.
Penn Family and Internal Medicine Longwood	1.00	.	1.00	.
Penn Family Medicine New Garden	1.00	.	1.00	.
Penn Family Medicine Phoenixville	1.00	.	1.00	.
Penn Family Medicine Unionville	2.15	0.3554	2.80	0.2896
Penn Family and Internal Medicine Cherry Hill	1.00	.	1.00	.
Penn Presbyterian Internal Medicine	1.00	.	1.00	.
Penn Internal Medicine East Marshall Street	1.00	.	1.00	.
Penn Primary Care and Integrative Medicine Whiteland	1.00	.	1.00	.
Penn Medicine Rittenhouse	1.00	.	1.00	.
Penn Medicine Bala Cynwyd	1.00	.	1.00	.
Penn Medicine Woodbury Heights	1.00	.	1.00	.
Penn Family Medicine Valley Forge	1.00	.	1.00	.
Penn Medicine Bucks County	1.00	.	1.00	.
Penn Family Medicine Limerick	1.00	.	1.00	.
Penn Family Medicine Gibbsboro	1.00	.	1.00	.
Penn Spruce Internal Medicine	1.00	.	1.00	.
Penn Family Medicine University City	1.00	.	1.00	.
Penn Family Medicine West Chester	1.00	.	1.00	.
Clinic appointment time				
8AM	Ref		Ref	
9AM	0.89	0.0617	0.91	0.1443
10AM	0.73	<.0001	0.81	0.0007
11AM	0.55	<.0001	0.64	<.0001
12PM	0.60	<.0001	0.63	<.0001
1PM	0.64	<.0001	0.73	<.0001
2PM	0.65	<.0001	0.71	<.0001
3PM	0.68	<.0001	0.78	0.0003
4PM	0.49	<.0001	0.60	<.0001
5PM	0.53	<.0001	0.49	<.0001
Month				

© 2019 Hsiang EY et al. *JAMA Network Open*.

September	Ref		Ref	
January	1.17	0.0319	1.04	0.6341
February	1.30	0.0005	1.00	0.9932
March	1.41	<.0001	1.08	0.3130
April	1.31	0.0004	0.97	0.6733
May	1.34	0.0004	0.92	0.3305
June	1.28	0.0018	1.05	0.5332
July	1.23	0.0136	0.86	0.0774
August	1.31	0.0019	1.15	0.0948
October	1.19	0.0044	1.19	0.0050
November	1.20	0.0075	1.00	0.9541
December	1.08	0.2874	0.94	0.4140
Visit type				
Return patient visit	Ref		Ref	
New patient visit	0.84	<.0001	0.88	0.0048

© 2019 Hsiang EY et al. *JAMA Network Open*.

eTable 7. Regression Table for Conditional Logistic Clinic Appointment Time Model With Continuous Appointment Time Variables for Breast Cancer Screening

Variable	Clinician Ordered Test		Patient Completed Test	
	Odds Ratio	*P* value	Odds Ratio	*P* value
Appointment year				
9/1/2014-8/31/2015	Ref		Ref	
9/1/2015-8/31/2016	1.13	0.0033	1.06	0.1840
Age at visit	1.00	0.1110	1.01	0.0004
Race				
White	Ref		Ref	
Asian/East Indian	1.16	0.1259	1.28	0.0093
Black	1.28	<.0001	1.19	0.0005
Hispanic	1.81	<.0001	1.69	<.0001
Other	0.95	0.4932	1.01	0.9257
Insurance				
Private	Ref		Ref	
Medicaid	0.64	<.0001	.83	0.0097
Medicare	0.76	<.0001	.89	0.0114
Charlson comorbidity index	0.89	<.0001	.93	<.0001
Income				
Less than $50,000	Ref		Ref	
$50,000 to $100,000	0.97	0.6139	1.00	0.9473
Greater than $100,000	0.92	0.1974	1.04	0.5693
Missing	0.66	0.0090	.79	0.1554
Practice site				
Penn Internal Medicine - Michael Cirigliano Internal Medicine	Ref		Ref	
Penn Family Medicine Chestnut Hill	1.00	.	1.00	.
Delancey Medicine Associates	1.00	.	1.00	.
Penn Family and Internal Medicine Lincoln	1.00	.	1.00	.
Penn Family Medicine Southern Chester County	1.00	.	1.00	.
Penn Internal Medicine University City	1.45	0.5583	3.47	0.1238
Penn General Internal Medicine Perelman	1.00	.	1.00	.
Penn Center for Primary Care, Penn Presbyterian Medical Center	1.00	.	1.00	.
Penn Radnor Internal Medicine	0.86	0.4943	.91	0.6606

© 2019 Hsiang EY et al. *JAMA Network Open*.

Penn Radnor Internal Medicine Geriatrics	1.00	.	1.00	.
Penn Internal Medicine Media	1.00	.	1.00	.
Penn Internal Medicine Mayfair	1.49	0.3468	1.99	0.2193
Penn Internal Medicine - J. Edwin Wood Clinic	1.00	.	1.00	.
Penn Family Medicine Kennett	1.00	.	1.00	.
Penn Family Care	1.00	.	1.00	.
Penn Family and Internal Medicine Longwood	1.00	.	1.00	.
Penn Family Medicine New Garden	1.00	.	1.00	.
Penn Family Medicine Phoenixville	1.00	.	1.00	.
Penn Family Medicine Unionville	1.99	0.4035	2.72	0.3035
Penn Family and Internal Medicine Cherry Hill	1.00	.	1.00	.
Penn Presbyterian Internal Medicine	1.00	.	1.00	.
Penn Internal Medicine East Marshall Street	1.00	.	1.00	.
Penn Primary Care and Integrative Medicine Whiteland	1.00	.	1.00	.
Penn Medicine Rittenhouse	1.00	.	1.00	.
Penn Medicine Bala Cynwyd	1.00	.	1.00	.
Penn Medicine Woodbury Heights	1.00	.	1.00	.
Penn Family Medicine Valley Forge	1.00	.	1.00	.
Penn Medicine Bucks County	1.00	.	1.00	.
Penn Family Medicine Limerick	1.00	.	1.00	.
Penn Family Medicine Gibbsboro	1.00	.	1.00	.
Penn Spruce Internal Medicine	1.00	.	1.00	.
Penn Family Medicine University City	1.00	.	1.00	.
Penn Family Medicine West Chester	1.00	.	1.00	.
Appointment hour	0.94	<.0001	.95	<.0001
Month				
September	Ref		Ref	
January	1.17	0.0276	1.04	0.5963
February	1.31	0.0003	1.01	0.9358
March	1.42	<.0001	1.09	0.2833
April	1.33	0.0002	.98	0.7528
May	1.35	0.0003	.93	0.3875
June	1.30	0.0011	1.06	0.4754
July	1.24	0.0111	.86	0.0871
August	1.31	0.0014	1.16	0.0812
October	1.20	0.0027	1.20	0.0034

© 2019 Hsiang EY et al. *JAMA Network Open*.

November	1.21	0.0044	1.01	0.8969
December	1.09	0.2393	.95	0.4780
Visit type				
Return patient visit	Ref		Ref	
New patient visit	0.84	<.0001	.88	0.0058

© 2019 Hsiang EY et al. *JAMA Network Open*.

eTable 8. Regression Table for Conditional Logistic Clinic Appointment Time Model With Hourly Appointment Time Variables for Colorectal Cancer Screening

**Variable**	**Clinician Ordered Test**		**Patient Completed Test**	
	**Odds Ratio**	***P* value**	**Odds Ratio**	***P* value**
Appointment year				
9/1/2014-8/31/2015	Ref		Ref	
9/1/2015-8/31/2016	1.43	<.0001	1.14	<.0001
Age at visit	0.96	<.0001	.98	<.0001
Gender				
Male	Ref		Ref	
Female	0.83	<.0001	.87	<.0001
Race				
White				
Asian/East Indian	0.90	0.1984	1.00	0.9903
Black	1.06	0.1674	1.06	0.1728
Hispanic	0.93	0.5122	.84	0.1172
Other	0.93	0.2149	.83	0.0013
Insurance				
Private	Ref		Ref	
Medicaid	0.58	<.0001	.84	0.0040
Medicare	0.67	<.0001	.91	0.0258
Charlson comorbidity index	0.92	<.0001	.98	0.0004
Income				
Less than $50,000	Ref		Ref	
$50,000 to $100,000	1.04	0.3759	1.04	0.4292
Greater than $100,000	1.14	0.0177	1.15	0.0117
Missing	1.08	0.5753	.74	0.0266
Practice site				

© 2019 Hsiang EY et al. *JAMA Network Open*.

Penn Internal Medicine - Michael Cirigliano Internal Medicine	Ref		Ref	
Penn Family Medicine Chestnut Hill	1.00	.	1.00	.
Delancey Medicine Associates	1.00	.	1.00	.
Penn Family and Internal Medicine Lincoln	1.00	.	1.00	.
Penn Family Medicine Southern Chester County	1.00	.	1.00	.
Penn Internal Medicine University City	0.84	0.6999	.98	0.9575
Penn General Internal Medicine Perelman	1.00	1.0000	1.00	.
Penn Center for Primary Care, Penn Presbyterian Medical Center	0.62	0.7521	1.01	0.9904
Penn Radnor Internal Medicine	0.72	0.1744	1.17	0.4512
Penn Radnor Internal Medicine Geriatrics	1.00	.	1.00	.
Penn Internal Medicine Media	1.00	.	1.00	.
Penn Internal Medicine Mayfair	1.06	0.8697	1.00	0.9914
Penn Internal Medicine - J. Edwin Wood Clinic	1.00	.	1.00	.
Penn Family Medicine Kennett	1.00	.	1.00	.
Penn Family Care	1.00	.	1.00	.
Penn Family and Internal Medicine Longwood	1.00	.	1.00	.
Penn Family Medicine New Garden	1.00	.	1.00	.
Penn Family Medicine Phoenixville	1.00	.	1.00	.
Penn Family Medicine Unionville	0.18	0.0217	1.01	0.9794
Penn Family and Internal Medicine Cherry Hill	1.00	.	1.00	.
Penn Presbyterian Internal Medicine	1.00	.	1.00	.
Penn Internal Medicine East Marshall Street	1.00	.	1.00	.
Penn Primary Care and Integrative Medicine Whiteland	1.00	.	1.00	.
Penn Medicine Rittenhouse	1.00	.	1.00	.
Penn Medicine Bala Cynwyd	1.00	.	1.00	.
Penn Medicine Woodbury Heights	1.00	.	1.00	.
Penn Family Medicine Valley Forge	1.00	.	1.00	.
Penn Medicine Bucks County	1.00	.	1.00	.
Penn Family Medicine Limerick	1.00	.	1.00	.
Penn Family Medicine Gibbsboro	1.00	.	1.00	.

© 2019 Hsiang EY et al. *JAMA Network Open*.

Penn Spruce Internal Medicine	1.00	.	1.00	.
Penn Family Medicine University City	1.00	.	1.00	.
Penn Family Medicine West Chester	1.00	.	1.00	.
Clinic appointment time				
8AM	Ref		Ref	
9AM	0.91	0.0783	.86	0.0023
10AM	0.78	<.0001	.88	0.0071
11AM	0.64	<.0001	.77	<.0001
12PM	0.56	<.0001	.76	0.0017
1PM	0.68	<.0001	.72	<.0001
2PM	0.64	<.0001	.73	<.0001
3PM	0.66	<.0001	.80	<.0001
4PM	0.50	<.0001	.74	<.0001
5PM	0.54	<.0001	.60	<.0001
Month				
September	Ref		Ref	
January	1.30	<.0001	1.24	0.0002
February	1.45	<.0001	1.21	0.0015
March	1.62	<.0001	1.12	0.0641
April	1.61	<.0001	1.18	0.0093
May	1.48	<.0001	1.18	0.0098
June	1.64	<.0001	1.23	0.0016
July	1.49	<.0001	1.24	0.0010
August	1.80	<.0001	1.28	0.0002
October	1.13	0.0230	1.03	0.5369
November	1.24	0.0003	1.04	0.5390

© 2019 Hsiang EY et al. *JAMA Network Open*.

December	1.14	0.033<br>7	1.08	0.1865
Visit type				
Return patient visit	Ref		Ref	
New patient visit	1.01	0.875<br>4	1.00	0.9483

© 2019 Hsiang EY et al. *JAMA Network Open*.

eTable 9. Regression Table for Conditional Logistic Clinic Appointment Time Model With Continuous Appointment Time Variables for Colorectal Cancer Screening

**Variable**	**Clinician Ordered Test**		**Patient Completed Test**	
	**Odds Ratio**	***P* value**	**Odds Ratio**	***P* value**
Appointment year				
9/1/2014-8/31/2015	Ref		Ref	
9/1/2015-8/31/2016	1.43	<.0001	1.14	<.0001
Age at visit	.96	<.0001	.98	<.0001
Gender				
Male	Ref		Ref	
Female	.82	<.0001	.87	<.0001
Race				
White	Ref		Ref	
Asian/East Indian	.90	0.1992	1.00	0.9864
Black	1.06	0.1864	1.06	0.1799
Hispanic	.94	0.6004	.84	0.1131
Other	.93	0.2316	.83	0.0013
Insurance				
Private	Ref		Ref	
Medicaid	.57	<.0001	.84	0.0026
Medicare	.67	<.0001	.91	0.0148
Charlson comorbidity index	.92	<.0001	.98	0.0004
Income				
Less than $50,000	Ref		Ref	
$50,000 to $100,000	1.05	0.3242	1.04	0.3880
Greater than $100,000	1.15	0.0132	1.15	0.0108
Missing	1.09	0.5362	.74	0.0234
Practice site				
Penn Internal Medicine - Michael Cirigliano Internal Medicine	Ref		Ref	
Penn Family Medicine Chestnut Hill	1.00	.	1.00	.
Delancey Medicine Associates	1.00	.	1.00	.
Penn Family and Internal Medicine Lincoln	1.00	.	1.00	.
Penn Family Medicine Southern Chester County	1.00	.	1.00	.
Penn Internal Medicine University City	.78	0.5717	.97	0.9478
Penn General Internal Medicine Perelman	1.00	.	1.00	.

© 2019 Hsiang EY et al. *JAMA Network Open*.

Penn Center for Primary Care, Penn Presbyterian Medical Center	.59	0.7188	1.01	0.9913
Penn Radnor Internal Medicine	.78	0.2929	1.18	0.4134
Penn Radnor Internal Medicine Geriatrics	1.00	.	1.00	.
Penn Internal Medicine Media	1.00	.	1.00	.
Penn Internal Medicine Mayfair	1.10	0.7914	1.00	0.9956
Penn Internal Medicine - J. Edwin Wood Clinic	1.00	.	1.00	.
Penn Family Medicine Kennett	1.00	.	1.00	.
Penn Family Care	1.00	.	1.00	.
Penn Family and Internal Medicine Longwood	1.00	.	1.00	.
Penn Family Medicine New Garden	1.00	.	1.00	.
Penn Family Medicine Phoenixville	1.00	.	1.00	.
Penn Family Medicine Unionville	.16	0.0193	1.01	0.9870
Penn Family and Internal Medicine Cherry Hill	1.00	.	1.00	.
Penn Presbyterian Internal Medicine	1.00	.	1.00	.
Penn Internal Medicine East Marshall Street	1.00	.	1.00	.
Penn Primary Care and Integrative Medicine Whiteland	1.00	.	1.00	.
Penn Medicine Rittenhouse	1.00	.	1.00	.
Penn Medicine Bala Cynwyd	1.00	.	1.00	.
Penn Medicine Woodbury Heights	1.00	.	1.00	.
Penn Family Medicine Valley Forge	1.00	.	1.00	.
Penn Medicine Bucks County	1.00	.	1.00	.
Penn Family Medicine Limerick	1.00	.	1.00	.
Penn Family Medicine Gibbsboro	1.00	.	1.00	.
Penn Spruce Internal Medicine	1.00	.	1.00	.
Penn Family Medicine University City	1.00	.	1.00	.
Penn Family Medicine West Chester	1.00	.	1.00	.
Appointment hour	.94	<.0001	.97	<.0001
Month				
September	Ref		Ref	
January	1.30	<.0001	1.25	0.0002
February	1.47	<.0001	1.21	0.0012
March	1.63	<.0001	1.13	0.0535
April	1.63	<.0001	1.18	0.0069
May	1.49	<.0001	1.19	0.0080
June	1.65	<.0001	1.23	0.0013
July	1.50	<.0001	1.25	0.0008

© 2019 Hsiang EY et al. *JAMA Network Open*.

August	1.82	<.0001	1.29	0.0001
October	1.14	0.0159	1.04	0.4669
November	1.25	0.0002	1.04	0.4900
December	1.15	0.0186	1.09	0.1548
Visit type				
Return patient visit	Ref		Ref	
New patient visit	1.00	0.8880	.99	0.8510

© 2019 Hsiang EY et al. *JAMA Network Open*.

eTable 10. Regression Table for Generalized Estimated Equations Model Clustered on Primary Care Physician With Hourly Appointment Time Variables for Breast Cancer Screening

**Variable**	**Clinician Ordered Test**		**Patient Completed Test**	
	**Odds Ratio**	***P* value**	**Odds Ratio**	***P* value**
Appointment year				
9/1/2014-8/31/2015	Ref		Ref	
9/1/2015-8/31/2016	1.11	0.0328	1.01	0.7597
Age at visit	1.00	0.2600	1.01	0.0005
Race				
White	Ref		Ref	
Asian/East Indian	1.27	0.0110	1.34	0.0066
Black	1.31	<.0001	1.22	<.0001
Hispanic	1.73	<.0001	1.72	0.0001
Other	.94	0.3805	.99	0.8856
Insurance				
Private	Ref		Ref	
Medicaid	.61	<.0001	.77	0.0003
Medicare	.75	<.0001	.84	0.0008
Charlson comorbidity index	.89	<.0001	.93	<.0001
Income				
Less than $50,000	Ref		Ref	
$50,000 to $100,000	.98	0.7065	1.02	0.7396
Greater than $100,000	.93	0.2410	1.07	0.2740
Missing	.60	0.0003	.76	0.1096
Practice site				
Penn Internal Medicine - Michael Cirigliano Internal Medicine	Ref		Ref	
Penn Family Medicine Chestnut Hill	.44	0.0819	.32	0.0019
Delancey Medicine Associates	.82	0.3831	.35	<.0001
Penn Family and Internal Medicine Lincoln	.39	0.1143	.40	0.0284
Penn Family Medicine Southern Chester County	.07	<.0001	.06	<.0001
Penn Internal Medicine University City	.76	0.1909	.58	0.0001
Penn General Internal Medicine Perelman	.41	<.0001	.19	<.0001
Penn Center for Primary Care, Penn Presbyterian Medical Center	.54	0.0014	.37	<.0001

© 2019 Hsiang EY et al. *JAMA Network Open*.

Penn Radnor Internal Medicine	.53	<.0001	.44	<.0001
Penn Radnor Internal Medicine Geriatrics	.65	<.0001	.46	<.0001
Penn Internal Medicine Media	.19	<.0001	.13	<.0001
Penn Internal Medicine Mayfair	.51	0.0257	.39	0.0010
Penn Internal Medicine - J. Edwin Wood Clinic	.56	0.0048	.32	<.0001
Penn Family Medicine Kennett	.61	0.0711	.45	0.0007
Penn Family Care	.36	<.0001	.29	<.0001
Penn Family and Internal Medicine Longwood	.33	<.0001	.27	<.0001
Penn Family Medicine New Garden	.09	<.0001	.10	<.0001
Penn Family Medicine Phoenixville	.86	0.5275	.19	<.0001
Penn Family Medicine Unionville	.23	0.0084	.24	0.0003
Penn Family and Internal Medicine Cherry Hill	.29	<.0001	.09	<.0001
Penn Presbyterian Internal Medicine	.71	0.3205	.39	<.0001
Penn Internal Medicine East Marshall Street	.40	<.0001	.34	<.0001
Penn Primary Care and Integrative Medicine Whiteland	.36	0.0091	.20	<.0001
Penn Medicine Rittenhouse	.61	0.0303	.40	<.0001
Penn Medicine Bala Cynwyd	.48	0.0056	.30	<.0001
Penn Medicine Woodbury Heights	.18	<.0001	.12	<.0001
Penn Family Medicine Valley Forge	.42	<.0001	.36	<.0001
Penn Medicine Bucks County	.41	<.0001	.33	<.0001
Penn Family Medicine Limerick	.21	<.0001	.17	<.0001
Penn Family Medicine Gibbsboro	.33	<.0001	.17	<.0001
Penn Spruce Internal Medicine	.94	0.7795	.41	<.0001
Penn Family Medicine University City	.43	<.0001	.27	<.0001
Penn Family Medicine West Chester	1.08	0.4956	.58	<.0001
Appointment hour				
8AM	Ref		Ref	
9AM	.90	0.0692	.91	0.1296
10AM	.74	<.0001	.79	0.0005
11AM	.56	<.0001	.63	<.0001
12PM	.65	0.0003	.65	0.0001
1PM	.65	<.0001	.71	<.0001
2PM	.66	<.0001	.70	<.0001
3PM	.66	<.0001	.74	<.0001
4PM	.51	<.0001	.58	<.0001
5PM	.54	<.0001	.48	<.0001

© 2019 Hsiang EY et al. *JAMA Network Open*.

Month				
September	Ref		Ref	
January	1.20	0.0123	1.04	0.5682
February	1.31	0.0003	1.01	0.8555
March	1.44	<.0001	1.10	0.2358
April	1.27	0.0033	.94	0.4824
May	1.35	0.0002	.94	0.4685
June	1.29	0.0023	1.06	0.5228
July	1.23	0.0158	.87	0.1193
August	1.24	0.0158	1.09	0.3266
October	1.19	0.0026	1.19	0.0021
November	1.21	0.0033	1.02	0.8027
December	1.11	0.1456	.96	0.5918
Visit type				
Return patient visit	Ref		Ref	
New patient visit	.79	0.0001	.83	0.0003
Intercept	3.43	<.0001	1.17	0.4860

© 2019 Hsiang EY et al. *JAMA Network Open*.

eTable 11. Regression Table for Generalized Estimated Equations Model Clustered on Primary Care Physician With Continuous Appointment Time Variables for Breast Cancer Screening

**Variable**	**Clinician Ordered Test**		**Patient Completed Test**	
	**Odds Ratio**	***P* value**	**Odds Ratio**	***P* value**
Appointment year				
9/1/2014-8/31/2015	Ref		Ref	
9/1/2015-8/31/2016	1.11	0.0307	1.02	0.7403
Age at visit	1.00	0.2812	1.01	0.0006
Race				
White	Ref		Ref	
Asian/East Indian	1.28	0.0086	1.34	0.0057
Black	1.30	<.0001	1.22	<.0001
Hispanic	1.75	<.0001	1.74	0.0001
Other	.94	0.3779	.99	0.8909
Insurance				
Private	Ref		Ref	
Medicaid	.60	<.0001	.77	0.0002
Medicare	.74	<.0001	.84	0.0004
Charlson comorbidity index	.89	<.0001	.93	<.0001
Income				
Less than $50,000	Ref		Ref	
$50,000 to $100,000	.98	0.6318	1.01	0.7849
Greater than $100,000	.93	0.2194	1.07	0.2900
Missing	.61	0.0004	.77	0.1232
Practice site				
Penn Internal Medicine - Michael Cirigliano Internal Medicine	Ref		Ref	
Penn Family Medicine Chestnut Hill	.45	0.0908	.33	0.0024
Delancey Medicine Associates	.83	0.4046	.35	<.0001
Penn Family and Internal Medicine Lincoln	.41	0.1338	.42	0.0353
Penn Family Medicine Southern Chester County	.07	<.0001	.06	<.0001
Penn Internal Medicine University City	.78	0.2349	.59	0.0004
Penn General Internal Medicine Perelman	.42	<.0001	.19	<.0001
Penn Center for Primary Care, Penn Presbyterian Medical Center	.55	0.0017	.38	<.0001
Penn Radnor Internal Medicine	.52	<.0001	.44	<.0001
Penn Radnor Internal Medicine Geriatrics	.64	<.0001	.45	<.0001

Penn Internal Medicine Media	.19	<.0001	.13	<.0001
Penn Internal Medicine Mayfair	.51	0.0303	.39	0.0011
Penn Internal Medicine - J. Edwin Wood Clinic	.59	0.0077	.33	<.0001
Penn Family Medicine Kennett	.61	0.0760	.46	0.0009
Penn Family Care	.38	<.0001	.30	<.0001
Penn Family and Internal Medicine Longwood	.34	<.0001	.28	<.0001
Penn Family Medicine New Garden	.10	<.0001	.10	<.0001
Penn Family Medicine Phoenixville	.88	0.5886	.19	<.0001
Penn Family Medicine Unionville	.23	0.0101	.25	0.0004
Penn Family and Internal Medicine Cherry Hill	.30	<.0001	.09	<.0001
Penn Presbyterian Internal Medicine	.72	0.3248	.39	<.0001
Penn Internal Medicine East Marshall Street	.42	<.0001	.35	<.0001
Penn Primary Care and Integrative Medicine Whiteland	.36	0.0103	.20	<.0001
Penn Medicine Rittenhouse	.60	0.0269	.39	<.0001
Penn Medicine Bala Cynwyd	.49	0.0084	.31	<.0001
Penn Medicine Woodbury Heights	.19	<.0001	.12	<.0001
Penn Family Medicine Valley Forge	.43	<.0001	.36	<.0001
Penn Medicine Bucks County	.41	<.0001	.33	<.0001
Penn Family Medicine Limerick	.22	<.0001	.17	<.0001
Penn Family Medicine Gibbsboro	.32	<.0001	.17	<.0001
Penn Spruce Internal Medicine	.97	0.9055	.42	<.0001
Penn Family Medicine University City	.45	<.0001	.28	<.0001
Penn Family Medicine West Chester	1.14	0.2439	.60	<.0001
Appointment hour	.94	<.0001	.95	<.0001
Month				
September	Ref		Ref	
January	1.21	0.0098	1.05	0.5374
February	1.31	0.0003	1.02	0.8219
March	1.44	<.0001	1.10	0.2364
April	1.28	0.0024	.95	0.5160
May	1.36	0.0001	.95	0.5124
June	1.30	0.0017	1.06	0.4718
July	1.24	0.0116	.87	0.1301
August	1.24	0.0125	1.09	0.3073
October	1.20	0.0017	1.19	0.0016
November	1.22	0.0024	1.02	0.7549
December	1.11	0.1286	.96	0.6470

© 2019 Hsiang EY et al. *JAMA Network Open*.

Visit type				
Return patient visit	Ref		Ref	
New patient visit	.80	0.0002	.83	0.0003
Intercept	3.19	<.0001	1.12	0.5800

© 2019 Hsiang EY et al. *JAMA Network Open*.

eTable 12. Regression Table for Generalized Estimated Equations Model Clustered Around Primary Care Physician With Hourly Appointment Time Variables for Colorectal Cancer Screening

**Variable**	**Clinician Ordered Test**		**Patient Completed Test**	
	**Odds Ratio**	***P* value**	**Odds Ratio**	***P* value**
Appointment year				
9/1/2014-8/31/2015	Ref		Ref	
9/1/2015-8/31/2016	1.36	<.0001	1.12	0.0005
Age at visit	.97	<.0001	.98	<.0001
Gender				
Male	Ref		Ref	
Female	.96	0.4245	.94	0.0308
Race				
White	Ref		Ref	
Asian/East Indian	1.02	0.8383	1.02	0.7635
Black	1.08	0.1123	1.07	0.1435
Hispanic	1.04	0.7010	.86	0.1557
Other	.97	0.5444	.83	0.0009
Insurance				
Private	Ref		Ref	
Medicaid	.57	<.0001	.78	0.0003
Medicare	.67	<.0001	.89	0.0037
Charlson comorbidity index	.92	<.0001	.98	0.0005
Income				
Less than $50,000	Ref		Ref	
$50,000 to $100,000	1.05	0.2528	1.08	0.1083
Greater than $100,000	1.15	0.0206	1.20	0.0037
Missing	1.00	0.9784	.75	0.0456
Practice site				
Penn Internal Medicine - Michael Cirigliano Internal Medicine	Ref		Ref	
Penn Family Medicine Chestnut Hill	.15	<.0001	.32	<.0001
Delancey Medicine Associates	.52	<.0001	.41	<.0001
Penn Family and Internal Medicine Lincoln	.01	<.0001	.27	<.0001
Penn Family Medicine Southern Chester County	.03	<.0001	.20	<.0001
Penn Internal Medicine University City	.56	<.0001	.63	<.0001
Penn General Internal Medicine Perelman	24.04	<.0001	.68	<.0001

© 2019 Hsiang EY et al. *JAMA Network Open*.

Penn Center for Primary Care, Penn Presbyterian Medical Center	.40	<.0001	.56	<.0001
Penn Radnor Internal Medicine	.79	0.1811	.66	<.0001
Penn Radnor Internal Medicine Geriatrics	1.75	<.0001	.57	<.0001
Penn Internal Medicine Media	.22	<.0001	.36	<.0001
Penn Internal Medicine Mayfair	.19	<.0001	.48	0.0016
Penn Internal Medicine - J. Edwin Wood Clinic	.21	<.0001	.32	<.0001
Penn Family Medicine Kennett	.13	0.0010	.37	<.0001
Penn Family Care	.31	<.0001	.51	<.0001
Penn Family and Internal Medicine Longwood	.19	<.0001	.41	0.0299
Penn Family Medicine New Garden	.07	<.0001	.25	<.0001
Penn Family Medicine Phoenixville	.31	0.0010	.24	<.0001
Penn Family Medicine Unionville	.08	0.0166	.23	<.0001
Penn Family and Internal Medicine Cherry Hill	.13	<.0001	.32	<.0001
Penn Presbyterian Internal Medicine	.54	0.0129	.55	0.0004
Penn Internal Medicine East Marshall Street	.07	<.0001	.27	<.0001
Penn Primary Care and Integrative Medicine Whiteland	.21	0.0219	.44	<.0001
Penn Medicine Rittenhouse	.06	<.0001	.35	<.0001
Penn Medicine Bala Cynwyd	.24	<.0001	.35	<.0001
Penn Medicine Woodbury Heights	.05	<.0001	.18	<.0001
Penn Family Medicine Valley Forge	.30	<.0001	.51	<.0001
Penn Medicine Bucks County	.21	<.0001	.22	<.0001
Penn Family Medicine Limerick	.16	<.0001	.20	<.0001
Penn Family Medicine Gibbsboro	.01	<.0001	.21	<.0001
Penn Spruce Internal Medicine	.42	<.0001	.46	<.0001
Penn Family Medicine University City	.35	<.0001	.41	<.0001
Penn Family Medicine West Chester	.12	0.0064	.38	<.0001
Appointment hour				
8AM	Ref		Ref	
9AM	.98	0.7029	.89	0.0166
10AM	.84	0.0008	.89	0.0108
11AM	.70	<.0001	.79	<.0001
12PM	.67	<.0001	.82	0.0396
1PM	.73	<.0001	.75	<.0001
2PM	.68	<.0001	.76	<.0001
3PM	.69	<.0001	.83	0.0008
4PM	.53	<.0001	.76	<.0001

© 2019 Hsiang EY et al. *JAMA Network Open*.

5PM	.60	<.0001	.62	<.0001
Month				
September	Ref		Ref	
January	1.35	<.0001	1.30	<.0001
February	1.41	<.0001	1.22	0.0010
March	1.53	<.0001	1.12	0.0621
April	1.58	<.0001	1.17	0.0086
May	1.43	<.0001	1.19	0.0169
June	1.55	<.0001	1.20	0.0020
July	1.41	<.0001	1.26	<.0001
August	1.68	<.0001	1.26	0.0008
October	1.16	0.0033	1.04	0.4169
November	1.26	0.0003	1.04	0.4561
December	1.17	0.0254	1.10	0.1291
Visit type				
Return patient visit	Ref		Ref	
New patient visit	1.01	0.8058	.95	0.1667
Intercept	15.44	<.0001	2.58	<.0001

© 2019 Hsiang EY et al. *JAMA Network Open*.

eTable 13. Regression Table for Generalized Estimated Equations Model Clustered Around Primary Care Physician With Continuous Appointment Time Variables for Colorectal Cancer Screening

**Variable**	**Clinician Ordered Test**		**Patient Completed Test**	
	**Odds Ratio**	***P* value**	**Odds Ratio**	***P* value**
Appointment year				
9/1/2014-8/31/2015	Ref		Ref	
9/1/2015-8/31/2016	1.36	<.0001	1.12	0.0006
Age at visit	.97	<.0001	.98	<.0001
Gender				
Male	Ref		Ref	
Female	.96	0.3923	.93	0.0235
Race				
White	Ref		Ref	
Asian/East Indian	1.02	0.7858	1.02	0.7499
Black	1.08	0.1226	1.07	0.1468
Hispanic	1.04	0.6844	.86	0.1526
Other	.97	0.5430	.83	0.0009
Insurance				
Private	Ref		Ref	
Medicaid	.57	<.0001	.78	0.0002
Medicare	.67	<.0001	.89	0.0022
Charlson comorbidity index	.92	<.0001	.98	0.0004
Income				
Less than $50,000	Ref		Ref	
$50,000 to $100,000	1.05	0.2552	1.08	0.0982
Greater than $100,000	1.14	0.0217	1.21	0.0034
Missing	1.01	0.9559	.75	0.0457
Practice site				
Penn Internal Medicine - Michael Cirigliano Internal Medicine	Ref		Ref	
Penn Family Medicine Chestnut Hill	.16	<.0001	.32	<.0001
Delancey Medicine Associates	.52	<.0001	.41	<.0001
Penn Family and Internal Medicine Lincoln	.01	<.0001	.27	<.0001
Penn Family Medicine Southern Chester County	.03	<.0001	.20	<.0001
Penn Internal Medicine University City	.57	<.0001	.63	<.0001

© 2019 Hsiang EY et al. *JAMA Network Open*.

Penn General Internal Medicine Perelman	23.95	<.0001	.68	<.0001
Penn Center for Primary Care, Penn Presbyterian Medical Center	.41	<.0001	.56	<.0001
Penn Radnor Internal Medicine	.79	0.1966	.66	<.0001
Penn Radnor Internal Medicine Geriatrics	1.74	<.0001	.56	<.0001
Penn Internal Medicine Media	.22	<.0001	.36	<.0001
Penn Internal Medicine Mayfair	.19	<.0001	.47	0.0013
Penn Internal Medicine - J. Edwin Wood Clinic	.21	<.0001	.32	<.0001
Penn Family Medicine Kennett	.13	0.0010	.37	<.0001
Penn Family Care	.32	<.0001	.52	<.0001
Penn Family and Internal Medicine Longwood	.20	<.0001	.41	0.0302
Penn Family Medicine New Garden	.07	<.0001	.25	<.0001
Penn Family Medicine Phoenixville	.31	0.0012	.24	<.0001
Penn Family Medicine Unionville	.08	0.0175	.23	<.0001
Penn Family and Internal Medicine Cherry Hill	.14	<.0001	.32	<.0001
Penn Presbyterian Internal Medicine	.54	0.0153	.55	0.0003
Penn Internal Medicine East Marshall Street	.07	<.0001	.27	<.0001
Penn Primary Care and Integrative Medicine Whiteland	.21	0.0225	.43	<.0001
Penn Medicine Rittenhouse	.06	<.0001	.35	<.0001
Penn Medicine Bala Cynwyd	.24	<.0001	.35	<.0001
Penn Medicine Woodbury Heights	.05	<.0001	.18	<.0001
Penn Family Medicine Valley Forge	.30	<.0001	.51	<.0001
Penn Medicine Bucks County	.21	<.0001	.22	<.0001
Penn Family Medicine Limerick	.16	<.0001	.20	<.0001
Penn Family Medicine Gibbsboro	.01	<.0001	.21	<.0001
Penn Spruce Internal Medicine	.43	<.0001	.47	<.0001
Penn Family Medicine University City	.36	<.0001	.41	<.0001
Penn Family Medicine West Chester	.13	0.0073	.39	<.0001
Appointment hour	.94	<.0001	.97	<.0001
Month				
September	Ref		Ref	
January	1.35	<.0001	1.30	<.0001
February	1.42	<.0001	1.22	0.0011
March	1.53	<.0001	1.12	0.0613
April	1.59	<.0001	1.17	0.0080

© 2019 Hsiang EY et al. *JAMA Network Open*.

May	1.43	<.0001	1.19	0.0170
June	1.55	<.0001	1.20	0.0021
July	1.42	<.0001	1.26	<.0001
August	1.69	<.0001	1.26	0.0007
October	1.17	0.0027	1.04	0.4119
November	1.26	0.0002	1.04	0.4555
December	1.17	0.0227	1.10	0.1294
Visit type				
Return patient visit	Ref		Ref	
New patient visit	1.02	0.7525	.95	0.1481
Intercept	15.94	<.0001	2.53	<.0001

© 2019 Hsiang EY et al. *JAMA Network Open*.

eFigure 1. Exclusions for the Patient Sample in Breast Cancer Screening

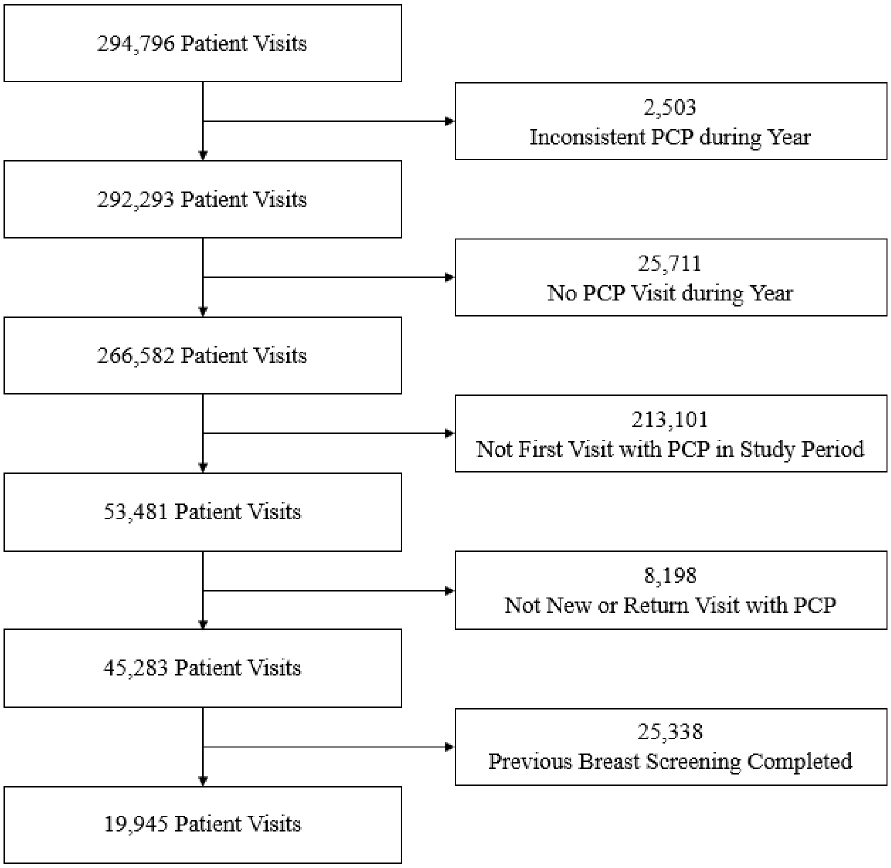

© 2019 Hsiang EY et al. *JAMA Network Open*.

eFigure 2. Exclusions for the Patient Sample in Colorectal Cancer Screening

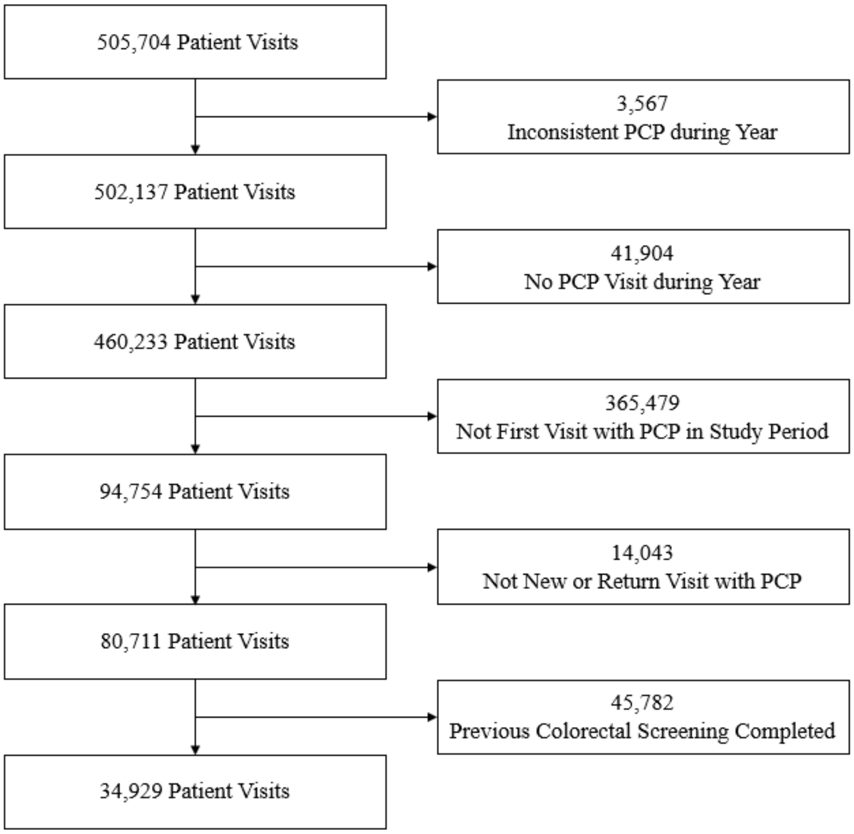

© 2019 Hsiang EY et al. *JAMA Network Open*.
